# Vitamin Supplementation and Dementia: A Systematic Review

**DOI:** 10.3390/nu14051033

**Published:** 2022-02-28

**Authors:** Victoria Gil Martínez, Ana Avedillo Salas, Sonia Santander Ballestín

**Affiliations:** 1 Department of Geriatric Care, Bonifatius Hospital Lingen, 49808 Lingen, Germany; victoriiagil@gmail.com; 2 Department of Pharmacology, Physiology and Legal and Forensic Medicine, Faculty of Medicine, University of Zaragoza, 50009 Zaragoza, Spain; aave60@hotmail.com

**Keywords:** dementia, mild cognitive impairment, vitamins, B complex vitamins, vitamin D, vitamin E

## Abstract

Background: Dementia is a syndrome characterized by progressive cognitive impairment that interferes with independent function in daily activities. Symptoms of dementia depend on its cause and vary greatly between individuals. There is extensive evidence supporting a relationship between diet and cognitive functions. This systematic review studies the efficacy of using vitamin supplements in the diet as a solution to nutritional deficiencies and the prevention of dementia and mild cognitive impairment. Methods: An intensive search of different databases (PubMed, Web of Science, and Cochrane CENTRAL) was performed. Articles that were published between 2011 and November 2021 were retrieved using the mentioned search strategy. This systematic review has been conducted according to the PRISMA statement. Results: Folic acid supplementation proved to have better outcomes on cognitive tests than their respective control groups. The combined supplementation of folic acid and vitamin B12 showed some discrepancies between studies. Thiamine as supplementation did not only prove to have a positive impact on cognitive performance when given alone but also when given in combination with folic acid. Regarding vitamin D supplementation, the results observed were not so encouraging. A concomitant supplementation of low-dose vitamin E and vitamin C was also not associated with an improvement of cognitive function. Conclusions: The findings of this systematic review suggest that supplementation of B Complex vitamins, especially folic acid, may have a positive effect on delaying and preventing the risk of cognitive decline. Ascorbic acid and a high dose of vitamin E, when given separately, also showed positive effects on cognitive performance, but there is not sufficient evidence to support their use. The results of vitamin D supplementation trials are not conclusive in assessing the potential benefits that vitamin D might have on cognition.

## 1. Introduction

### 1.1. Dementia and Mild Cognitive Impairment: Definition and Clinical Characteristics

Dementia is a syndrome characterized by progressive cognitive impairment beyond what would be expected from natural aging itself that interferes with the ability to function independently in day-to-day activities [1,2]. Although there is no doubt that the prevalence and incidence of dementia exponentially rise with increasing age, not everyone develops it [3].

According to the latest revision of the International Classification of Diseases or ICD-11, which will be effective from 1 January 2022, dementia is defined as: “an acquired brain syndrome characterized by a decline from a previous level of cognitive functioning”. This definition also adds that “based on available evidence, the cognitive impairment is attributed or assumed to be attributable to a neurological or medical condition that affects the brain, trauma, nutritional deficiency, chronic use of specific substances or medications, or exposure to heavy metals or other toxins” [4].

Symptoms of dementia depend on its cause and vary greatly between individuals, but some of them include memory loss; concentration difficulties; changes in personality, mood, and behavior; agnosia (inability to recognize objects); apraxia (inability to perform previously learned tasks); aphasia (inability to comprehend or formulate language); agraphia (inability to communicate through writing) and alexia (loss of the ability to read) [5,6]. The latest update of the Diagnostic and Statistical Manual of Mental Disorders (DSM-V) replaced the term dementia with the term “major neurocognitive disorder” and proposed a series of criteria to diagnose it [7].

Firstly, it requires a substantial decline from a previous level of performance in one or more cognitive domains, these being complex attention, executive function, learning and memory, language, perceptual-motor, and social cognition. Secondly, the cognitive impairment must be enough to interfere with independence in everyday activities. Thirdly, the cognitive deficits must not occur exclusively in the context of delirium. Finally, they cannot be better explained by another mental disorder (for example, major depressive disorder or schizophrenia) [7].

Prior to the onset of dementia, there can be a prodromal neurological syndrome known as mild cognitive impairment (MCI) [8]. To be considered MCI, the difficulties experienced in cognitive areas like language, memory, thinking, or judgement need to be greater than the cognitive changes expected by normal individuals as they age but not severe enough to constitute dementia [9].

### 1.2. Subtypes of Dementia

The concept of dementia includes a series of subtypes that are characterized by their underlying pathology. The four main ones are Alzheimer’s disease, vascular dementia, frontotemporal lobar degeneration, and Lewy body dementia [10]. The main features of each of these subtypes are detailed in Table 1 [5,10,11,12,13,14,15,16,17].

### 1.3. Risk Factors

There are multiple risk factors that can contribute to the development of dementia. Some, such as advanced age, genetic factors, or family history, cannot be modified. However, there are others that, if modified, can reduce the likelihood of developing dementia or at least delay its onset [18].

In 2017, a review by the Lancet Commission gathered compelling evidence on nine potentially modifiable risk factors for dementia: less education, hypertension, hearing impairment, smoking, obesity, depression, physical inactivity, diabetes, and low social contact [19].

In 2020, an update of this review added three new risk factors to the list: excessive alcohol consumption, traumatic brain injury, and air pollution [20].

These findings have also been verified by other institutions such as the World Health Organization (WHO). In 2019, the WHO published a series of recommendations to reduce the risk of cognitive decline and dementia, including smoking cessation, treatment of high blood pressure and diabetes, as well as encouraging physical activity [18].

Other risk factors such as diet or nutritional deficits are shown to have an influence on the development of the disease. Hence, nutritional supplementation may be a possible solution to prevent cognitive decline [21,22]. Because of this, it is important to update vitamin deficits and their implication on dementia.

Vitamins are organic substances that play an important role in the proper functioning of human physiology, as well as in various basic metabolic pathways that support fundamental cellular functions [23]. Despite their great importance, they are synthetized in very small quantities in the human body. That is why we require a regular supply in the diet [24].

As diet and nutrition are potentially modifiable contributors to age-associated cognitive decline, more research is being done to investigate this [25].

Various vitamins and other dietary compounds have been studied on cognitive decline and dementia [26].

### 1.4. Justification and Aim

Dementia is not only one of the main causes of disability in older adults, but it also significantly increases dependency, economic overload, and psychological stress on the caregiver [27].

In May 2017, the WHO recognized dementia as a public health priority, endorsing the Global Action Plan on the Public Health Response to Dementia 2017–2025, which defines a set of actions to promote dementia awareness and research; facilitate the diagnosis, treatment, and support for caregivers and families; and suggest measures to reduce the risk of dementia [28]. The idea is that the Member States set ambitious responses to achieve these objectives [28].

Globally, the prevalence of dementia in 2018 was 50 million [29], a figure estimated to reach 82 million by 2030 [18] and 152 million by 2050 [29]. It is expected to rise particularly in low-income and middle-income countries [29].

It is extremely important that countries focus on reducing potentially modifiable risk factors for dementia due to the increase in the prevalence of dementia [18], its significant social and economic impact [18], and the absence of curative treatment.

There is extensive evidence supporting the relationship between diet and vitamin supplementation on cognitive function. On the one hand, some authors as Suh S. et al. [30] or Forbes S. et al. [31] have already investigated the potential benefits of vitamins on cognition. However, these studies only analyze the effects of vitamin supplementation in patients who have not yet developed dementia. On the other hand, there are other published systematic reviews that also study the efficacy of vitamin supplementation in participants with mild cognitive impairment or dementia, such as in the studies from Li S. et al. [32] and Farina N. et al. [33]. However, these studies only analyze the influence of one type of vitamin.

During our search, we could also find some reviews that assess the efficacy of different vitamins and nutritional supplements; however, they were mostly narrative reviews [34,35]. For this reason, this systematic review is necessary to analyze all vitamins involved in cognitive deterioration and dementia in both people with and without dementia.

## 2. Materials and Methods

### 2.1. Search Strategy

The search was made using PubMed, Web of Science, and Cochrane CENTRAL. The studies were identified by combining the words “cognition” and “mild cognitive impairment” with the following keywords: “vitamins”, “vitamin A”, “vitamin B1”, “vitamin B2”, “vitamin B3”, “vitamin B5”, “vitamin B6”, “vitamin B9”, “vitamin B12”, “vitamin C”, “vitamin D”, “vitamin E”, “vitamin K”, “vitamin H”, or their respective synonyms. A detailed search strategy is shown in Appendix A.

**Figure 1:**
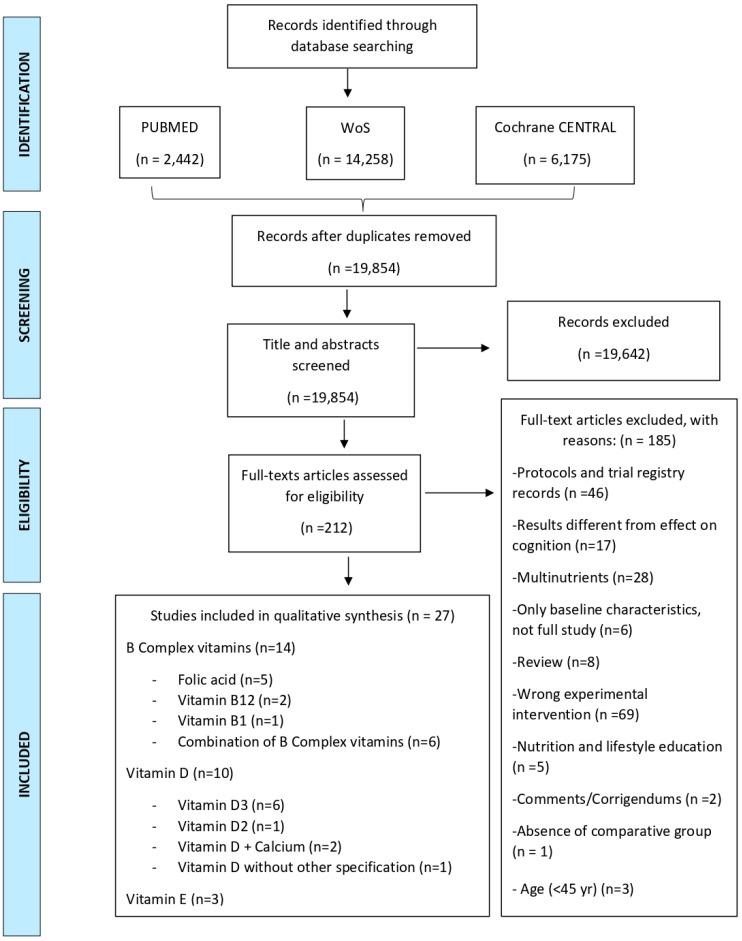
PRISMA 2009 Flow diagram illustrating the study selection process [36]. CENTRAL: Cochrane Controlled Register of Trials, WoS: Web of Science.

MeSH terms (Medical Subject Headings) and the Boolean operators “AND” and “OR” were used in the search. The study was conducted according to the PRISMA statement [36]. Articles published until November 2021 were retrieved. After removing duplicates, titles and abstracts were screened excluding those that did not meet the inclusion criteria. The remaining records were assessed for eligibility by careful review of their full texts. A flow chart illustrating the study selection process is shown in Figure 1.

### 2.2. Inclusion Criteria

The inclusion criteria applied in this systematic review were proposed according to the PICOS algorithm (Table 2).

### 2.3. Exclusion Criteria

The proposed exclusion criteria for this systematic review were (a) studies with insufficient data; (b) in vitro, in silico, or in vivo animal studies; (c) comments, expert opinions, case reports, letters to the editor, reviews, protocols, and trial registry records; (d) studies that do not include at least one of the vitamins evaluated in this systematic review as an intervention; (e) studies published before 2011; and (f) studies in languages different from Spanish, English, or German.

### 2.4. Data Collection and Analysis

From each of the included studies, we extracted some information regarding the methodological characteristics, interventions, and participant characteristics.

The methodological characteristics are as followed: (a) clinical trial registration number, (b) author, (c) publication date, (d) trial design (randomized control trial or observational study), and (e) number of participants (recruited, allocated, evaluated).

The intervention characteristics are as followed: (a) type of intervention, (b) dosage regimen, (c) frequency of treatment, (d) duration of treatment, (e) concomitant treatments, and (f) type of control used (placebo, non-intervention, standard of care, and other interventions (including but not limited to vitamins)).

The participant’s characteristics are as followed: (a) cognitive status (dementia, Alzheimer’s disease, MCI), (b) other concomitant pathologies, (c) ethnicity, (d) baseline age (mean, median), and (e) gender.

Some of this information has been detailed in Appendix B.

## 3. Results

### 3.1. Search Results and Description of Studies

Articles were retrieved according to the mentioned search strategy and published from 2011 until November 2021 were retrieved. In total, we retrieved 22,875 articles. After removing duplicates, 19,854 records remained. Then, we conducted a screening of their respective titles and abstracts. We finally excluded 19,642 records. The full texts of the remaining 212 records were then carefully reviewed and assessed for eligibility. Finally, we were able to include 27 articles in this systematic review that met the inclusion criteria. The detailed process is depicted in Figure 1**.**

In order to analyze the results, we decided to group the studies into the type of intervention used. Regarding B Complex vitamins, we included a total of 14 studies. In total, eight of them compared B Complex vitamins to placebo [37,38,39,40,41,42,43,44], five of them compared B Complex vitamins to conventional treatment [45,46,47,48,49], and one compared vitamin B supplementation with vitamin C supplementation [50].

Regarding vitamin D, 10 studies were finally included. A total of 4 compared vitamin D supplementation with placebo [51,52,53,54], 4 compared the supplementation of vitamin D with non-intervention or conventional treatment [55,56,57,58], and 2 of the studies compared two different dosage regimens of vitamin D with each other [59,60].

As for vitamin E, only three studies were included. All of them compared vitamin E supplementation with placebo [61,62,63].

Finally, no studies regarding the supplementation of vitamin A and K met the inclusion criteria of this systematic review.

### 3.2. Analysis of Results

#### 3.2.1. Vitamin B Complex

The 14 included studies analyzed different B vitamins: vitamin B12 (two studies) [43,50], vitamin B1 (benfothiamine) (one study) [40], folic acid (five studies) [37,41,45,46,47], and the remaining six trials studied the concomitant use of various B Complex vitamins [38,39,42,44,48,49].

##### Comparison 1: B Complex Vitamins vs. Placebo

Intervention: All eight studies included in this comparison were placebo-controlled. However, the dosage regimen and type of vitamin B supplemented vary among the studies.

- Participants in Chen H. et al. received 1.2 mg folic acid and 50 µg vitamin B12 once daily for six months [39];
- People in the study by Dangour A. et al. received 1 mg vitamin B12 p.o (orally) daily for 12 months [43];
- In the trial by Walker J. et al., participants received 400 µg folic acid and 100 µg vitamin B12 supplementation p.o daily for 24 months [38];
- Participants in the RCT (Randomized controlled trial) by Kwok T. et al. were treated with 500 µg methylcobalamin and 400 µg folic acid p.o once daily for 24 months [42];
- Participants in Moore K. et al. received 400 µg folic acid, 10 µg vitamin B12, 10 mg vitamin B6, and 10 mg riboflavin over two years [44].
- Gibson G. et al. used, as intervention, a supplement of 300 mg benfotiamine b.i.d (twice a day) for 12 months [40];
- In the RCT by Chen H. et al., those in the intervention group received donepezil and a supplement of 1.25 mg folic acid daily for six months, and those in the control group only received placebo apart from donepezil [37];
- Finally, the study by Li M. et al. compared three intervention groups with placebo. The first IG received 800 µg folic acid plus 800 mg docosahexaenoic (DHA) p.o daily. The second IG only received 800 µg folic p.o daily and the third IG received 800 mg DHA p.o daily. The duration of the three treatments was six months [41].

Outcomes:

In each of these studies, different cognitive scales were used to assess the impact of vitamin supplementation on cognitive function.

Some of the RCTs showed very promising results regarding the use of B Complex vitamin supplementation. Those receiving folic acid in the study by Chen H. et al. showed statistically significant higher Mini Mental State Examination (MMSE) scores than the control group after six months of treatment (*p* = 0.041), proving that folic acid is beneficial in patients with Alzheimer’s disease [37]. However, there was no significant difference between both groups regarding Activities of Daily Living (ADL) scores after six months of treatment (*p* = 0.895) [37]. The detailed scores are shown in Table 3.

The combined use of folic acid and vitamin B12 may be linked to a lower risk of cognitive decline [38]. After 24 months of supplementation, a statistically significant increase from baseline to 24 months was detected, not only in the TICS-M total scores (modified telephone interview for cognitive status) (*p* = 0.032) but more specifically in the TICS-M immediate recall (*p* = 0.046) and delayed recall (*p* = 0.013) scores of those receiving folic acid and vitamin B12 in comparison to placebo [38]. For other TICS-M domains, no statistically significant changes were recorded [38].

Another RCT that studied the concomitant supplementation of folic acid and vitamin B12 was by Chen H. et al. [39]. Here, the cognitive decline was assessed using the Montreal Cognitive Assessment test (MoCA) and the Alzheimer’s Disease Assessment Scale-Cognitive Subscale (ADAS-Cog) [39]. Those in the intervention group proved to have a statistically significant increase in MoCA total scores (*p* = 0.029) as well as MoCA naming scores (*p* = 0.013) and MoCA orientation scores (*p* = 0.004) after six months of treatment when compared to placebo [39]. Other domains, such as MoCA visuospatial/executive abilities (*p* = 0.207), attention (*p* = 0.446), or language scores (*p* = 0.877) did not show statistically significant differences [39]. The interaction effect in ADAS-Cog attention scores was statistically significant (β [95%CI]: −0.675 [−1.162, −0.188] *p* = 0.008) [39]. This indicates that treatment over time was associated with a decrease in ADAS-Cog attention scores [39]. Total ADAS-Cog scores as well as other ADAS-Cog domains like registration, language, or executive abilities showed no statistically significant differences between participants in both groups [39].

Another study that showed encouraging outcomes regarding the changes in ADAS-Cog was by Gibson et al. [40] Here, those receiving benfotiamine had a lower increase in ADAS-Cog after 12 months of treatment than those treated with a placebo, therefore showing less cognitive decline [40]. However, it needs to be noted that the changes were only nearly statistically significant (*p* = 0.125) [40].

What did show statistically significant differences that favored the intervention group, was the mean change from baseline to twelve months in global Clinical Dementia Rating (CDR) (*p* = 0.034) and the Neuropsychiatric Inventory (NPI) in males at nine months (*p* = 0.014) and twelve months (*p* = 0.035) [40]. Nevertheless, no significant changes were registered on the Buschke Selective Reminding Test (SRT) as well as on the scores on the Alzheimer’s Disease Cooperative Study-Activities of Daily Living (ADCS-ADL) [40].

Daily oral supplementation of folic acid, DHA, or their combination also proved to significantly improve cognitive function when compared to placebo after six months of treatment [41]. In this RCT, the cognitive function was evaluated by the full-scale intelligence quotient (FSIQ) scores and index scores of the Chinese version of the Wechsler Adult Intelligence Scale-Revised (WAIS-RC) [41]. The WAIS-RC includes 11 subtests: Information, Similarities, Vocabulary, Comprehension, Arithmetic, Digit Span, Block Design, Picture Completion, Digit Symbol-Coding, Object Assembly, and Picture Arrangement [47].

With regard to FSIQ scores, only the combination group (Folic acid plus DHA) showed statistically significant improvements (*p* < 0.001) compared to the control group, while those receiving only folic acid did not show significant differences (*p* = 0.221) [41]. However, regarding some domains of the WAIS-RC, like the digit span scores or block design, those receiving a monotherapy folic acid proved to have significantly better scores (*p* = 0.001 and *p* = 0.0013, respectively) [41]. It has been detailed in Table 4.

Most of the trials included in this systematic review that analyze the concomitant supplementation of vitamin B12 and folic acid show very promising results. There are studies such as in Kwok T. et al. where this double therapy does not significantly attenuate cognitive decline [42]. In this study, participants were aged ≥65 years with MCI and elevated levels of homocysteine ≥10 µmol/L at baseline [42]. The mean changes in the Clinical Dementia Rating Sum of Boxes (CDR-SOB) over 24 months were 0.36 (95%CI 0.15–0.57) and 0.22 (95%CI 0.05–0.39) for the intervention and control groups, respectively [42].

Finally, there were two other trials that showed no evidence of a significant improvement in cognitive function after B Complex supplementation [43,44]. The RCT comparing vitamin B12 with placebo [43] in patients with moderate vitamin B12 deficiency and a baseline MMSE ≥ 24, showed very small changes in the California Verbal Learning Test (CVLT) in both supplementation and control groups [43]. The RCT by Moore et al. compared a quadruple supplementation (folic acid, vitamin B12, vitamin B6, and riboflavin) in generally healthy adults aged ≥70 years with a placebo. Here, vitamin B Complex supplementation appeared to have no significant impact on either frontal lobe or global cognitive function as measured by the Frontal Assessment Battery (FAB) and Repeatable Battery for the Assessment of Neuropsychological Status (RBANS) *p* = 0.485 and *p* = 0.117, respectively [44]. This has been detailed in Table 5.

##### Comparison 2: B Complex Vitamins vs. Conventional Treatment

Intervention: Five studies [45,46,47,48,49] compared supplementation of B Complex vitamins with conventional treatment.

- Participants in Ma F. et al. received 400 µg folic acid daily for six months [45];
- In another study by Ma F. et al., those in the intervention group also received 400 µg folic acid p.o daily, but in this case for twelve months [46];
- There was another study published by Ma. F. et al. where those in the intervention group also received 400 µg folic acid p.o daily. The duration of the treatment in this trial was 24 months [47];
- In the trial by Lu R. et al., participants received 90 mg thiamine and 30 mg folic acid daily for 96 weeks [49];
- Jiang B. et al. used, as an intervention, a supplement of 5 mg folic acid daily plus 500 µg vitamin B12 t.i.d. for 24 weeks [48].

Outcomes:

The three studies by Ma F. et al. used the full-scale intelligence quotient (FSIQ) scores and index scores of the Chinese version of the Wechsler Adult Intelligence Scale-Revised (WAIS-RC) to assess the cognitive function of Chinese older adults with MCI [45,46,47].

Daily oral supplementation of 400 µg folic acid for six months was sufficient to improve general intellectual function (*p* = 0.031) and showed better outcomes in the “Digit Span” subtest (*p* = 0.009) and “Block Design” subtest (*p* = 0.036) compared to those in the control group from baseline to month six [45].

Other cognitive subdomains assessed by the WAIS-RC did not show statistically significant differences between intervention and control groups [45].

400 µg folic supplementation for a year also improved the performance at the FSIQ (*p* = 0.028), “Information” subtest (*p* = 0.031), and “Digit Span” subtest (*p* = 0.009) from baseline to month 12 in the intervention group compared to those allocated to conventional treatment [46]. The results of other WAIS-RC subtests were not significant [46].

Similar findings were detected by a supplementation of 400 µg folic for 24 months [47]. Those in the intervention group performed significantly better in the FSIQ (adjusted *p* = 0.021), VIQ or Verbal Intelligence Quotient (adjusted *p* = 0.031), “Information” subtest (adjusted *p* = 0.021), and “Digit Span” subtest (adjusted *p* = 0.009) after 24 months compared to those receiving conventional treatment [47].

Another study compared the double supplementation of folic acid and vitamin B12 in patients with vascular cognitive impairment and complicated with hyperhomocystinemia [48]. Here, the vitamin supplementation showed very promising results regarding a significant cognitive improvement assessed by MoCA test after 24 weeks compared to those in the control group (Mean MoCA scores ± SD at 24 weeks: 24.90 ± 1.79 and 23.20 ± 1.58 for intervention and control groups, respectively; *p* < 0.01) [48]. Within the intervention group, MoCA scores at 4 and 12 weeks did not show significant differences (*p* > 0.05), but at 24 weeks the score was significantly higher compared to the previous time points (*p* < 0.01) [48].

The last study included in this group of comparisons was by Lu R. et al., where thiamine in combination with folic acid was compared to a non-intervention group [49]. Cognitive scores were assessed using the MoCA test. As well as in the previous trial, participants of the intervention group performed better after 96 weeks of supplementation when compared to the control group (*p* < 0.001) [49]. It needs to be noted that the participants in this study were undergoing haemodialysis due to end-stage kidney disease [49].

##### Comparison 3: Vitamin B vs. Vitamin C

Intervention: One study compared supplementation of Methylcobalamin with vitamin C [50]. These participants were randomized to either 500 µg Methylcobalamin p.o daily or 50 mg ascorbic acid p.o daily. Both interventions lasted twelve weeks [50].

Outcomes:

In this study, only postmenopausal women with mild to moderate cognitive dysfunction were included [50]. Regarding methylcobalamin supplementation, it did not show any significant improvement on cognitive function between baseline and after treatment in none of the following MMSE domains: “delayed verbal recall”, “naming”, and “repetition” (*p* > 0.05, respectively) [50]. The only domain that showed statistically significant differences between baseline and end of treatment was “immediate recall” (*p* = 0.038) [50].

On the other hand, a twelve-week supplementation with ascorbic acid did show significant improvements in not only MMSE “immediate recall” (*p* = 0.035), but also in “delayed verbal recall” (*p* = 0.027), “naming” (*p* = 0.042), and “repetition” (*p* = 0.031) when compared to baseline [50]. Other MMSE parameters did not show significant differences throughout the experiment [50].

#### 3.2.2. Vitamin D

Of the 10 included studies, 6 were about vitamin D3 [51,53,55,58,59,60], 1 about vitamin D2 [52], 2 about the concomitant supplementation of vitamin D and calcium [54,57], and 1 study did not specify the type of vitamin analyzed [56].

##### Comparison 1: Vitamin D vs. Placebo

Intervention: The four RCT included in this comparison were placebo-controlled. However, there is variation in the dosage regimen and type of intervention between the studies.

- Participants in Aspell N. et al. received supplementation of 50 µg vitamin D3 daily for six months [51];
- Those allocated in the intervention group by Stein M. et al. were treated with a high dose of vitamin D2 (6000 IU) daily for eight weeks [52];
- Participants recruited to the intervention arm of the VITAL trial received 2000 IU vitamin D3 p.o. daily accompanied with fish oil supplements [53];
- Rossom R.C. et al. decided to explore the supplementation of 400 IU vitamin D3 and 1000 mg calcium carbonate daily [54].

Outcomes:

Short-term vitamin D3 supplementation did not have a significant impact on global cognitive function assessed by the MoCA test (*p* = 0.186) on patients aged ≥60 years without cognitive impairment at baseline [51]. Neither a significant effect on other domain-specific tasks of executive function (Trials Making Tasks A and B or TMTA and TMTB), nor on attention (SART-CoV or Sustained Attention to Response Task Coefficient of Variation) were detected (*p* = 0.467 and *p* = 0.893, respectively) [51].

Another short-term supplementation, in this case with a higher Dose of vitamin D2, also did not find benefits on cognitive function after eight weeks of supplementation in participants with mild to moderate AD as assessed by the ADAS-Cog scale before and after treatment (*p* = 0.45) [52].

The next study used data from two subsets of the VITAL Trial. One subset was the VITAL-Cog (NCT01669915) and another subset was the CTSC-Cog (included in an ancillary study of depression VITAL-DEP: NCT01696435) [53]. According to the results, vitamin D3 supplementation did not slow cognitive decline among generally healthy adults aged ≥60 years [53].

Rossom R.C. et al. decided to explore the supplementation of 400 IU vitamin D3 and 1000 mg calcium carbonate daily on women aged ≥65 years without cognitive impairment at baseline [54]. No significant differences were detected between intervention and control groups regarding incidence of dementia during a mean follow-up of 7.8 yr (HR = 1.11; 95%CI: 0.71–1.74; *p* = 0.64) as well as incidence of MCI (HR = 0.95; 95%CI = 0.72–1.25; *p* = 0.72) [54].

##### Comparison 2: Vitamin D vs. Non-Intervention or Conventional Treatment

Intervention: Four studies compared supplementation of vitamin D with conventional treatment or non-intervention.

- Firstly, in Anweiler C. et al., those in the intervention group received a supplementation of 800 IU vitamin D3 p.o daily or 100,000 IU p.o per month [55];
- The intervention arm by Lee Y. et al. explored the supplementation of 1000 IU vitamin D daily, accompanied with exercise programs for twelve weeks [56];
- Finally, Beauchet O. et al. allocated a total of 20 patients to an intervention of fortified yoghurts daily, that included 400 IU vitamin D3, as well as 800 mg calcium for three months [57];
- Bischoff-Ferrari H. et al. allocated 1076 participants to a 2000 IU vitamin D3 supplementation daily for three years [58].

Outcomes:

The studies comparing the vitamin D supplementation with either conventional treatment or non-intervention show some discrepancies. On the one hand, those in the study by Anweiler C. et al. who received a vitamin D supplementation had better outcomes at the MMSE, Cognitive Assessment Battery (CAB), and Frontal Assessment Battery (FAB) scores at the end of the treatment than those in the control group, the differences being statistically significant (*p* = 0.04, *p* = 0.03 and *p* = 0.04, respectively) [55], as detailed in Table 6.

Cognitive function also appeared to be improved after a twelve-week vitamin D supplementation [56]. However, cognitive function assessed by the MMSE before and after treatment not only improved in the intervention group (*p* = 0.004), but also in the control group (*p* = 0.019), thus making the intergroup difference not statistically significant (OR = 0.826; 95%CI = 0.651–1.047; *p* = 0.114) [56].

In the study by Beauchet O. et al., those allocated with fortified yoghurts with vitamin D3 and calcium achieved better outcomes on cognitive performance compared to the control group after three months [57]. At the end of the follow-up, MMSE scores were higher in the intervention group compared to the control group (*p* = 0.010) [57]. The time to perform the TMTB significantly shortened after supplementation in the intervention group (*p* = 0.035) [57].

On the other hand, a 2000 IU vitamin D3 supplementation for three years did not improve cognitive function among adults aged ≥70 years and without major comorbidities [58].

##### Comparison 3: Two Different Dosage Regimens of Vitamin D

Intervention: Two trials compared different dosage regimens of vitamin D with each other. Both trials were double-blind RCT.

- Castle M. et al. allocated the participants into three intervention groups. The first one received a 600 IU vitamin D3 supplementation for a year, the second one received a 2000 IU vitamin D3 supplementation for a year and the third group was treated with 4000 IU vitamin D3 supplementation for a year [59];
- Those in the intervention group by Schietzel S. et al. received 2000 IU vitamin D3 daily, whereas the vitamin D3 supplementation in those in the control group was 800 IU daily [60].

Outcomes:

In one of the studies, a dosage regimen of 2000 IU vitamin D3 showed better performance on visual and working memory and learning tests than a 600 IU vitamin D supplementation or a 4000 IU vitamin D supplementation [59]. For example, the total errors on “Paired Associates Learning (PAL)” was significantly lower in those receiving supplementation of 2000 IU vitamin D3 (*p* = 0.004) [59]. Moreover, the third intervention group (4000 IU vitamin D3) was associated with a slower reaction time compared to the first intervention group (600 IU supplementation) (*p* < 0.01), thus implying that a higher dose might negatively affect reaction time [59].

On the other hand, supplementation of 2000 IU vitamin D3 on 137 individuals aged ≥60 years and with a baseline MMSA ≥24 was not significantly superior compared to an 800 IU vitamin D3 supplementation [60]. At 24 months, the difference in the unadjusted mean MMSE scores between both groups was not significant (*p* = 0.44) [60].

#### 3.2.3. Vitamin E

Of the three included studies [61,62,63], one of them studied the concomitant supplementation of vitamin C and vitamin E versus placebo [63].

##### Comparison 1: Vitamin E vs. Placebo

Intervention: Two double-blind, placebo-controlled trials compared the supplementation of vitamin E with a placebo [61,62]. One of the RCT was transformed into a cohort study [61].

- In the PREADVISE trial, participants were allocated into either one of three intervention groups or a placebo group. Those in the first intervention group (IG1) received 400 IU vitamin E daily, those in the second intervention group (IG2) were treated with 200 µg selenium daily, and finally, those assigned to the third intervention group (IG3) received a combination of vitamin E and selenium [61].
- In the study by Dysken. M.W. et al., participants were also allocated into either one of three intervention groups or a placebo group. Those in the first intervention group (IG1) received 1000 IU α-tocopherol p.o., b.i.d. Those in the second intervention group (IG2) were treated with 10 mg memantine p.o., b.i.d. Finally, those assigned to the third intervention group (IG3) received a combination of α-tocopherol and memantine [62].

Outcomes:

Neither vitamin E, selenium supplementation, nor their combination were able to prevent dementia [61]. Incidence rates of dementia did not differ among the four study arms [61]. Regarding the hazard rates for incident dementia in the intervention arms, none were significantly lower compared to placebo [61]. The intervention group 1 had HR = 0.88; 95%CI (0.64–1.20); *p* = 0.41; intervention group 2 had HR = 0.83; 95%CI (0.61–1.13); *p* = 0.23; and in the case of intervention group 3, it had HR = 1.00; 95%CI (0.74–1.35); *p* = 0.98 [61].

Nevertheless, the study by Dysken. M.W. et al. observed that 2000 IU α-tocopherol was associated with a slower cognitive decline among patients with mild to moderate AD, as measured by the ADCS-ADL Inventory (*p* = 0.03) compared to a placebo [62]. The differences in the groups receiving memantine alone or in combination with α-tocopherol were not statistically significant [62].

##### Comparison 2: Vitamin E Plus Vitamin C vs. Placebo

Intervention: One double-blind RCT compared the double combination of 300 mg vitamin E daily with 400 mg vitamin C daily for one year and compared it with placebo [63].

Outcomes:

This double combination did not prove to enhance cognitive performance assessed by the MMSE after a year of treatment [63]. Mean MMSE scores were not significantly different between the intervention and control group at neither six months nor twelve months of intervention (*p* > 0.05) [63].

## 4. Discussion

Mild cognitive impairment (MCI) and dementia are considered one of the major public health problems nowadays, partly because of extended life expectancy and therefore an increased population with chronic pathologies [64].

Currently, the most common cause of progressive cognitive impairment is Alzheimer’s disease [65]. Dementia and MCI interfere greatly with daily activities [65], which not only has a negative impact on the patient itself but also on the caregivers. To date, no cure has been found for Alzheimer’s disease [65]. In order to slow the progress and delay the onset of cognitive decline, it is vital to identify the risk factors associated with cognitive impairment with the aim of developing effective preventive strategies [66]. Given the fact that diet and nutrition are considered one of the potential modifiable risk factors [66], this systematic review has aimed to identify trials that studied vitamin supplementation in order to reach conclusions about their efficacy in preventing or slowing cognitive decline.

This systematic review covers not only vitamin supplementation in healthy older individuals but also in participants who already suffered from dementia or MCI at the beginning of their trial.

Many of the studies included in this systematic review show encouraging results regarding vitamin B complex supplementation. More specifically, all the studies included that compared folic acid supplementation with either placebo or conventional treatment proved to have better outcomes on cognitive tests than their respective control groups [37,41,45,46,47].

When it comes to monotherapy with vitamin B12 supplementation vs. placebo or conventional treatment, only one trial met the inclusion criteria [43]. However, no benefits were detected over one year of supplementation. This may be due to the fact that the included participants were relatively healthy [43]. Moreover, the duration of the supplementation may have also been too short to detect any effects [43].

One study compared supplementation of methylcobalamin with vitamin C [50]. As well as in the study by Dangour [43] et al., vitamin B12 supplementation did not improve cognitive function significantly [50]. However, ascorbic acid did contribute to a significantly better cognitive performance after 12 weeks [50].

The combined supplementation of folic acid and vitamin B12 showed some discrepancies between studies. Despite that in three of the included trials, this double supplementation was associated with better performance on cognitive assessments like MoCa or ADAS-Cog [38,39,48], in two other trials, this intervention did not attenuate cognitive decline [42,44]. In one of them, the reason may have been because of the small sample size or the fact that the intervention group had better cognitive functions at baseline [42].

Thiamine as supplementation did not only prove to have a positive impact on cognitive performance when given alone [40] but also when given in combination with folic acid [49].

The results observed can be compared with those of other systematic reviews. For example, in the one by Mccleery. J. et al. where the effect of vitamin and mineral supplementation on cognitive function and incident dementia in people with MCI was evaluated, a 6-to-24-month vitamin B supplementation did not prove to have beneficial effects on cognition [67].

Regarding vitamin D supplementation, the results observed were not so encouraging. When compared to a placebo, none of the included trials showed a significant improvement in cognitive function in those who received vitamin D [51,52,53].

However, in the study by Anweiler. C. et al., where vitamin D3 supplementation was compared with non-intervention, those in the intervention group had better scores in the different cognitive assessment scales and proved to have better executive functioning compared to the control group [55].

The dosage regimen can also play an important role in the effectiveness of an intervention. That is why some studies have tried to compare different dosages of vitamin D to establish which one shows a cognitive benefit [59,60]. Nevertheless, there are discrepancies between studies. On the one hand, Castle et al. observed not only that those receiving 2000 IU vitamin D3 had a better performance on visual and working memory and learning test compared to those allocated to smaller and higher dosages, but also that a higher dosage (4000 IU vitamin D) could be associated with negative effects like a slower reaction time [59]. On the other hand, Schietzel et al. did not find any advantage of 2000 IU vitamin D supplementation when compared to a smaller dosage [60].

Discordant results were also detected in trials comparing a double supplementation of vitamin D and calcium with either placebo [54] or non-intervention [57]. While the incidence of dementia and MCI in the study by Rossom et al. did not significantly differ between groups [54], those receiving fortified yogurt with calcium and vitamin D3 in the study by Baeauchet et al. did improve cognitive function compared to control [57].

In accordance with what we observed, other systematic reviews have found mixed results across the literature regarding cognitive function by vitamin D supplementation, thus resulting in a lack of certainty in assessing its potential beneficial effects on cognition [68].

Finally, with regards to vitamin E, only three studies met the inclusion criteria of this systematic review [61,62,63]. This makes the generalization of the outcomes difficult. While a dose of 2000 IU vitamin E daily seemed to be associated with a slower cognitive decline [62], a dose of 400 IU did not significantly reduce dementia [61]. A concomitant supplementation of low-dose vitamin E and vitamin C was also not associated with an improvement in cognitive function [63].

Over the years, there have been some systematic reviews published that try to assess the efficacy of Vitamin E in patients with dementia. In 2000, a systematic review published by Cochrane could not detect evidence of the efficacy of vitamin E in the treatment or prevention of patients suffering from either Alzheimer’s disease or mild cognitive impairment [33]. Later, the same systematic review underwent some updates. The last version, from 2017, still does not find evidence of vitamin E efficacy on cognitive function, daily living activities, or disease severity in patients with Alzheimer’s disease or mild cognitive impairment when compared to placebo [33].

According to these findings, in another systematic review, none of the included studies regarding vitamin E detected a significant efficacy on cognitive outcomes in non-demented middle-aged and older adults [31].

### Limitations

This systematic review also has some potential limitations. Firstly, the exclusion of publications written in languages other than English, Spanish, or German could have introduced bias. Secondly, despite a very intensive search, relevant publications may have been missed. Thirdly, each study has used different scales or tests to assess cognitive impairment, making it difficult to compare them. Fourthly, only trials about vitamin D, B Complex vitamins, vitamin E, and vitamin C met the inclusion criteria, while no trial about vitamin A or K could be included. The results observed cannot be generalized to all vitamins.

## 5. Conclusions

The findings of this systematic review suggest that supplementation of B Complex vitamins, especially a supplementation of folic acid, may have a positive effect on delaying and preventing the risk of cognitive decline.

Ascorbic acid and a high dose of vitamin E also showed positive effects on cognitive performance. However, due to the small number of studies included in this systematic review about these vitamins, there is not sufficient evidence to support their use.

Regarding vitamin D supplementation, the findings observed vary vastly among trials. This results in a lack of certainty in assessing the potential benefits that vitamin D might have on cognition.

## Figures and Tables

**Table 1:** Epidemiology and clinical features of the 4 most common dementia subtypes.

**Alzheimer’s disease**	- Most common subtype of dementia (60–80% of all dementia cases) [10]; - Characterized by extracellular beta-amyloid deposits (beta-amyloid plaques) and intracellular neurofibrillary tangles (tau tangles) in the cerebral cortex and subcortical gray matter [11]; - It follows an insidious and progressive course [11]; - The most characteristic early clinical symptoms are short-term memory disorders (e.g., difficulty remembering recent conversations or events). Long-term memory is still preserved for a long time [12].
**Vascular dementia**	- A frequent cause of dementia (around 20% of all dementia cases) [10]; - It can have a sudden or a progressive onset [5]; - Vascular dementia is usually associated with cerebrovascular diseases like stroke and lacunar infarcts, hemorrhage, cardioembolism, as well as other comorbidities like hypertension and diabetes mellitus [5,13]. Both micro-and macroangiopathic changes play an important role in the pathogenesis of vascular dementia [13]; - Its heterogeneous symptomatology depends on the type and localisation of the vascular damage. Therefore, while cortical lesions usually lead to aphasia, apraxia, or epileptic seizures, subcortical lesions are linked to bradyphrenia, executive dysfunctions, urinary incontinence, and parkinsonism [14].
**Frontotemporal lobar degeneration**	- Most frequent cause of early-onset dementia (people under the age of 60) [10]; - It is an heterogeneous neurodegenerative disorder that includes some clinical variants [15]: (a) A behavioural variant (bvFTD) characterised by personality disorders like apathy, aggression, and agitation; (b) A language variant (primary progressive aphasia or PPA); (c) These patients can also suffer from motor deficits like amyotrophic lateral sclerosis (ALS), corticobasal syndrome (CBS), or progressive supranuclear palsy (PSP) [15].
**Lewy body dementia (LBD)**	- It is associated with an abnormal aggregation of alpha-synuclein in neurons (Lewy bodies) [12]; - The most characteristic features are fluctuating cognition and attentional impairment. A remission to near-normal cognitive function can occur spontaneously [16]; - Other core symptoms of LBD are recurrent visual hallucinations and spontaneous parkinsonism [17].

ALS: amyotrophic lateral sclerosis; bvFTD: behavioural variant of Frontotemporal lobar degeneration; CBS: corticobasal syndrome; LBD: Lewy body dementia; PPA: primary progressive aphasia, PSP: progressive supranuclear palsy.

**Table 2:** Inclusion criteria based on PICO algorithm.

**Patient (P)**	Adults with normal cognition, MCI, or Alzheimer’s disease with an age > 45 yr. There were no restrictions on sex, ethnicity, or severity of the cognitive impairment at baseline.
**Intervention (I)**	Vitamins as dietary supplements (A, B_1_, B_2_, B_3_, B_5_, B_6_, B_9_, B_12_, H, C, D, E, K). Co-interventions between vitamins were allowed.
**Comparison (C)**	Standard of care, no intervention, placebo, another dosage regimen, or other intervention (including but not limited to vitamins).
**Outcome (O)**	- Incidence of all-cause dementia or mild cognitive impairment; - Cognitive function measured by cognitive scales like MMSE, ADAS-Cog, WAIS-RC, TMT, MocA, ADL-Score, FAB-Score, or CDR.
**Type of studies (S)**	- Both randomized controlled clinical trials (RCTs) and observational studies were included; - Articles published in the last ten years (from 2011 to 2021) in Spanish, German, or English.

ADAS-Cog: Alzheimer’s Disease Assessment Scale-Cognitive Subscale. ADL: Activities of Daily Living, CDR: Clinical Dementia Rating Scale, FAB: Frontal Assessment Battery, MCI: mild cognitive impairment, MMSE: Mini Mental State Examination, MoCA: Montreal Cognitive Assessment, RCT: randomized controlled trial, TMT: Trail Making Test, WAIS-RC: Wechsler Adult Intelligence Scale-Revised in China, yr: years.

**Table 3:** Mean MMSE and ADL scores after 6 months of treatment [37].

	Intervention Group	Control Group	*p* Value
Mean MMSE Scores ± SD after 6 months of supplementation	18.72 ± 6.56	16.80 ± 8.26	0.041
Mean ADL scores ± SD after 6 months of treatment	32.93 ± 10.93	34.10 ± 14.15	0.895

ADL: Activities of Daily Living; MMSE: Mini Mental State Examination; SD: standard deviation.

**Table 4:** Mean FSIQ score at baseline and at 6 months in the first intervention group and in the control group [41].

	At Baseline	At 6 Months	*p* Value
Mean FSIQ score ± SD in IG1 (FA + DHA)	100.45 ± 3.90	104.04 ± 2.72	*p* < 0.001
Mean FSIQ score ± SD in CG	101.68 ± 4.18	102.63 ± 2.61	

CG: control group; DHA: docosahexaenoic acid; FA: folic acid; FSIQ: Full Scale Intelligence Quotient; IG1: intervention group 1; SD: standard deviation.

**Table 5:** Mean FAB and RBANS scores before and after treatment in intervention and control groups [44].

	Pre-Intervention	Post-Intervention	*p* Value
Mean FAB total score	IG (15.1) vs. CG (14.7)	IG (14.7) vs. CG (14.4)	*p* = 0.485
Mean RBANS total score	IG (93.4) vs. CG (93.3)	IG (97.8) vs. CG (95.5)	*p* = 0.117

IG: intervention group; CG: control group; FAB: Frontal Assessment Battery; RBANS: Repeatable Battery for the Assessment of Neuropsychological Status.

**Table 6:** MMSE, CAB, and FAB scores after treatment in intervention and control groups, median (IQR) [55].

	Intervention Group	Control Group	*p* Value
MMSE score after treatment, median (IQR)	28.0 (4.0)	24.0 (4.0)	*p* = 0.04
CAB score after treatment, median (IQR)	90 (12.0)	89 (6.0)	*p* = 0.03
FAB score after treatment, median (IQR)	16.0 (2.0)	15.0 (3.0)	*p* = 0.04

CAB: Cognitive Assessment Battery; FAB: Frontal Assessment Battery; IQR: Interquartile Range; MMSE: Mini Mental State Examination.

## Data Availability

Not applicable.

## Appendix A. Search Strategy

**Pubmed**

#1 (vitamins) OR (dietary supplements) OR (multivitamin) OR (vitamin*) OR (supple*) OR (diet*) OR (supplement*)
#2 (vitamin A) OR (retinol) OR (retinoic acid)
#3 (vitamin D) OR (cholecalciferol) OR (vitamin D3) OR (ergocalciferol) OR (vitamin D2) OR (toxiferol) OR (calcitriol) OR (calcitriol derivative)
#4 (vitamin E)
#5 (vitamin K group) OR (vitamin K) OR (phylloquinone) OR (vitamin K1) OR (phytomenadione) OR (phytonadione)
#6 (vitamin B complex) OR (vitamin B group) OR (vitamin B1) OR (thiamine)
#7 (vitamin B2) OR (riboflavin)
#8 (vitamin B3) OR (niacin) OR (niacinamide) OR (nicotinamide) OR (nicotinic acid)
#9 (vitamin B6) OR (pyridoxine) OR (pyridoxal) OR (pyridoxamine) OR (pyridoxal 5′ phosphate) OR (pyridoxamine 5′ phosphate) OR (pyridoxine 5′ phosphate)
#10 (vitamin B9) OR (folic acid)
#11 (vitamin B12) OR (cobalamins) OR (cyanocobalamin) OR (hydroxocobalamin) OR (methylcobalamin)
#12 (vitamin B5) OR (pantothenic acid)
#13 (biotin) OR (vitamin H)
#14 (vitamin C) OR (l-ascorbic acid) OR (ascorbic acid) OR (ascorbate)
#15 (mild cognitive impairment) OR (MCI) OR (AAMI) OR (age-associated memory impairment) OR (age consistent memory impairment) OR (ACMI) OR (age related cognitive decline) OR (ARCD) OR (cognitive impairment with no dementia) OR (CIND)
#16 (cognition) OR (cognition disorders) OR (memory) OR (memory disorders) OR (mental performance) OR (mental perform*) OR (memory) OR (executive function) OR (attention)
#17 #1 OR #2 OR #3 OR #4 OR #5 OR #6 OR #7 OR #8 OR #9 OR #10 OR #11 OR #12 OR #13 OR #14
#18 #15 OR #16
#19 #18 AND #17
#20 #19 Filters: Filters: Free full text, Clinical Study, Clinical Trial, Clinical Trial, Phase I, Clinical Trial, Phase II, Clinical Trial, Phase III, Clinical Trial, Phase IV, Comparative Study, Controlled Clinical Trial, Meta-Analysis, Randomized Controlled Trial, in the last 10 years, English, German, Spanish Sort by: Publication Date

**Web of Science**

#1 TS = ((vitamins) OR (dietary supplements) OR (multivitamin) OR (vitamin*) OR (supple*) OR (diet*) OR (supplement*))
#2 TS = ((vitamin A) OR (retinol) OR (retinoic acid))
#3 TS = ((vitamin D) OR (cholecalciferol) OR (vitamin D3) OR (ergocalciferol) OR (vitamin D2) OR (toxiferol) OR (calcitriol) OR (calcitriol derivative))
#4 TS = ((vitamin E))
#5 TS = ((vitamin K group) OR (vitamin K) OR (phylloquinone) OR (vitamin K1) OR (phytomenadione) OR (phytonadione))
#6 TS = ((vitamin B complex) OR (vitamin B group) OR (vitamin B1) OR (thiamine))
#7 TS = ((vitamin B2) OR (riboflavin))
#8 TS = ((vitamin B3) OR (niacin) OR (niacinamide) OR (nicotinamide) OR (nicotinic acid))
#9 TS = ((vitamin B6) OR (pyridoxine) OR (pyridoxal) OR (pyridoxamine) OR (pyridoxal 5′ phosphate) OR (pyridoxamine 5′ phosphate) OR (pyridoxine 5′ phosphate))
#10 TS = ((vitamin B9) OR (folic acid))
#11 TS = ((vitamin B12) OR (cobalamins) OR (cyanocobalamin) OR (hydroxocobalamin) OR (methylcobalamin))
#12 TS = ((vitamin B5) OR (pantothenic acid))
#13 TS = ((biotin) OR (vitamin H))
#14 TS = ((vitamin C) OR (l-ascorbic acid) OR (ascorbic acid) OR (ascorbate))
#15 TS = ((mild cognitive impairment) OR (MCI) OR (AAMI) OR (age-associated memory impairment) OR (age consistent memory impairment) OR (ACMI) OR (age related cognitive decline) OR (ARCD) OR (cognitive impairment with no dementia) OR (CIND))
#16 TS = ((cognition) OR (cognition disorders) OR (memory) OR (memory disorders) OR (mental performance) OR (mental perform*) OR (memory) OR (executive function) OR (attention))
#17 #1 OR #2 OR #3 OR #4 OR #5 OR #6 OR #7 OR #8 OR #9 OR #10 OR #11 OR #12 OR #13 OR #14
#18 #15 OR #16
#19 #18 AND #17
#20 #18 AND #17 and Open Access and 2022 or 2021 or 2020 or 2019 or 2018 or 2017 or 2016 or 2015 or 2014 or 2013 or 2012 or 2011 (Publication Years)
#21 #18 AND #17 and Open Access and 2022 or 2021 or 2020 or 2019 or 2018 or 2017 or 2016 or 2015 or 2014 or 2013 or 2012 or 2011 (Publication Years) and Articles (Document Types)

**Cochrane Central**

#1 MeSH descriptor: [Vitamins] explode all trees
#2 (vitamins) OR (dietary supplements) OR (multivitamin) OR (vitamin*) OR (supple*) OR (diet*) OR (supplement*)
#3 #1 OR #2
#4 MeSH descriptor: [Vitamin A] explode all trees
#5 (vitamin A) OR (retinol) OR (retinoic acid)
#6 #4 OR #5
#7 MeSH descriptor: [Vitamin D] explode all trees
#8 (vitamin D) OR (cholecalciferol) OR (vitamin D3) OR (ergocalciferol) OR (vitamin D2) OR (toxiferol) OR (calcitriol) OR (calcitriol derivative)
#9 #7 OR #8
#10 MeSH descriptor: [Vitamin E] explode all trees
#11 (vitamin E)
#12 #10 OR #11
#13 MeSH descriptor: [Vitamin K] explode all trees
#14 (vitamin K group) OR (vitamin K) OR (phylloquinone) OR (vitamin K1) OR (phytomenadione) OR (phytonadione)
#15 #13 OR #14
#16 MeSH descriptor: [Vitamin B Complex] explode all trees
#17 (vitamin B complex) OR (vitamin B group)
#18 #16 OR #17
#19 MeSH descriptor: [Thiamine] explode all trees
#20 (vitamin B1) OR (thiamine)
#21 #19 OR #20
#22 MeSH descriptor: [Riboflavin] explode all trees
#23 (vitamin B2) OR (riboflavin)
#24 #22 OR #23
#25 MeSH descriptor: [Niacinamide] explode all trees
#26 (vitamin B3) OR (niacin) OR (niacinamide) OR (nicotinamide) OR (nicotinic acid)
#27 #25 OR #26
#28 MeSH descriptor: [Vitamin B 6] explode all trees
#29 (vitamin B6) OR (pyridoxine) OR (pyridoxal) OR (pyridoxamine) OR (pyridoxal 5′ phosphate) OR (pyridoxamine 5′ phosphate) OR (pyridoxine 5′ phosphate)
#30 #28 OR #29
#31 MeSH descriptor: [Folic Acid] explode all trees
#32 (vitamin B9) OR (folic acid)
#33 #31 OR #32
#34 MeSH descriptor: [Vitamin B 12] explode all trees
#35 (vitamin B12) OR (cobalamins) OR (cyanocobalamin) OR (hydroxocobalamin) OR (methylcobalamin)
#36 #34 OR #35
#37 MeSH descriptor: [Pantothenic Acid] explode all trees
#38 (vitamin B5) OR (pantothenic acid)
#39 #37 OR #38
#40 MeSH descriptor: [Biotin] explode all trees
#41 (biotin) OR (vitamin H)
#42 #40 OR #41
#43 MeSH descriptor: [Ascorbic Acid] explode all trees
#44 (vitamin C) OR (l-ascorbic acid) OR (ascorbic acid) OR (ascorbate)
#45 #43 OR #44
#46 MeSH descriptor: [Cognitive Dysfunction] explode all trees
#47 (mild cognitive impairment) OR (MCI) OR (AAMI) OR (age-associated memory impairment) OR (age consistent memory impairment) OR (ACMI) OR (age related cognitive decline) OR (ARCD) OR (cognitive impairment with no dementia) OR (CIND)
#48 #46 OR #47
#49 MeSH descriptor: [Memory Disorders] explode all trees
#50 (cognition) OR (cognition disorders) OR (memory) OR (memory disorders) OR (mental performance) OR (mental perform*) OR (memory) OR (executive function) OR (attention)
#51 #49 OR #50
#52 #2 OR #5 OR #8 OR #11 OR #14 OR #17 OR #20 OR #23 OR #26 OR #29 OR #32 OR #35 OR #38 OR #41 OR #44
#53 #3 OR #6 OR #9 OR #12 OR #15 OR #18 OR #21 OR #24 OR #27 OR #30 OR #33 OR #36 OR #39 OR #42 OR #45
#54 #47 OR #50
#55 #48 OR #51
#56 #54 AND #52
#57 #55 AND #53
#58 #55 AND #53 with Publication Year from 2011 to 2021, in Trials

## Appendix B

**Table A1:** Characteristics of included studies on vitamins.

Reference/Register Number	Study Design/Population of Study	No. of Participants	Mean Age ± SD, Years	Sex
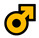
Vitamin B12 + Folic acid vs. Placebo				
Kwok T. et al. [42] CUHK_CCT00373	RCT (placebo-controlled)/People ≥65 yr with MCI and elevated levels of serum homocysteine ≥10 µmol/L	IG: *n* = 138; CG: *n* = 141	IG: 76.9 ± 5.4; CG: 78.0 ± 5.3	IG: 63.1%; CG: 56.1%
Vitamin B12 + Folic acid vs. Placebo				
Walker J. et al. [38] NCT00214682	RCT/Community-dwelling adults between 60–74 yr with elevated psychological distress (Kessler Distress 10-scale; score >15)	IG: *n* = 447; CG: *n* = 453	IG: 65.92 ± 4.3; CG: 65.97 ± 4.18	IG: 40.5%; CG: 39.1%
Vitamin B12 + Folic acid vs. Conventional treatment				
Jiang B. et al. [48]	RCT/Patients with vascular cognitive impairment-no dementia (VCIND), complicated with hyperhomocystinemia	IG: *n* = 60; CG: *n* = 60	Average age ±SD, years: 63 ± 1.87	Total, %: 65%
Vitamin B12 + Folic acid vs. Placebo				
Chen H. et al. [39] ChiCTR-IOR-16009731	RCT (single-blind, placebo-controlled, single-center, parallel-group)/Patients >45 yr diagnosed clinically as probable AD and in a stable condition (MoCA less than 22)	IG: *n* = 60; CG: *n* = 60	IG: 68.58 ± 7.29; CG: 68.02 ± 8.34	IG: 50%; CG: 43.33%
Vitamin B12 vs. Placebo				
Dangour A. et al. [43] ISRCTN54195799	RCT (double-blind, placebo-controlled)/People ≥75 yr with MMSE ≥ 24 and moderate vitamin B12 deficiency (serum vitamin B12 concentrations 107–210 pmol/L) and absence of anemia	IG: *n* = 99; CG: *n* = 102	IG: 79.9 ± 3.5; CG: 80.1 ± 3.7	IG: 46.5%; CG: 47.1%
Folic acid vs. Placebo				
Ma F. et al. [46]	RCT (single blind experimental design)/People ≥65 yr with MCI	IG: *n* = 84; CG: *n* = 84	IG: 73.71 ± 2.57; CG: 73.52 ± 3.03	IG: 32.14%; CG: 30.95%
Folic acid + DHA vs. Placebo				
Li M. et al. [41] Chi-CTR-IOR-16008351	RCT (double-blind, placebo-controlled, two-center)/Elderly with MCI ≥60 yr and absence of mental disorders	IG_1_: *n* = 60; IG_2_: *n* = 60; IG_3_: *n* = 60; CG: *n* = 60	IG_1_: 70.33 ± 7.7; IG_2_: 70.20 ± 6.13; IG_3_: 71.55 ± 6.62; CG: 70.38 ± 6.73	IG_1_: 40%; IG_2_: 40%; IG_3_: 40%; CG: 45%
Folic acid vs. Placebo				
Ma F. et al. [45] ChiCTR-TRC-13003227	RCT (single-center)/Chinese adults ≥65 yr with MCI who are unexposed to folic acid fortification	IG: *n* = 90; CG: *n* = 90	IG: 74.82 ± 2.75; CG: 74.63 ± 3.21	IG: 36.25%; CG: 34.18%
Folic acid vs. Conventional treatment				
Ma F. et al. [47] ChiCTR-TRC-13003227	RCT (single-center)/Chinese adults ≥65 yr with MCI	IG: *n* = 90; CG: *n* = 90	IG: 74.82 ± 2.75; CG: 74.63 ± 3.21	IG: 43.33%; CG: 42.22%
Folic acid + Donepezil vs. Placebo + Donepezil				
Chen H. et al. [37] ChiCTR-TRC-13003246	RCT (single-center, single-blind)/Patients with a new diagnosis of possible AD of mild or moderate severity (defined as an MMSE total score between 3 and 26) and currently being treated with Donepezil	IG: *n* = 61; CG: *n* = 60	IG: 68.10 ± 8.50; CG: 67.63 ± 7.92	IG: 54.10%; CG: 46.67%
Vitamin B1 (Benfotiamine) vs. Placebo				
Gibson G.E. et al. [40] NCT02292238	RCT (placebo-controlled, Phase IIa, double blind)/Amyloid positive patients ≥60 yr with amnestic MCI or mild dementia due to AD and MMSE > 21	IG: *n* = 34; CG: *n* = 36	IG: 75.74 ± 6.91; CG: 75.81 ± 7.19	IG: 32.4%; CG: 50%
Vitamin B1 (Thiamin) vs. Non Intervention				
Lu R. et al. [49] ChiCTR-IPR-17012210	RCT (single-center)/adults with end-stage kidney disease and cognitive impairment (MoCA score <26)	IG: *n* = 25; CG: *n* = 25	IG: 66.16 ± 7.61; CG: 69.00 ± 10.80	IG: 72%; CG: 76%
(Vitamin B12+ Vitamin B6 + Vitamin B2+ Folic acid) vs. Placebo				
Moore K. et al. [44]	RCT/Generally healthy older adults ≥70 yr	IG: *n* = 124; CG: *n* = 125	IG: 77.9 ± 4.2; CG: 78.2 ± 4.7	IG: 48.5%; CG: 41.1%
Vitamin B12 vs. Vitamin C				
Vijayakumar T.M. et al. [50] CTRI No: REF/2016/02/010726	RCT (double-blind, parallel-group)/Postmenopausal women (50–75 yr) with mild to moderate cognitive dysfunction	IG: *n* = 28; CG: *n* = 28	IG: 57.56 ± 7.72; CG: 55.88 ± 6.01	IG: 0%; CG: 0%
High Dose Vitamin D2 vs. Placebo				
Stein M. et al. [52] ACTRN12606000324516	RCT (double-blind)/Community-dwelling participants ≥60 yr with mild-moderate AD (MMSE score 12–24)	IG: *n* = 16; CG: *n* = 16	Median age [IQR],yr: IG: 75 [64.5–80] CG: 79 [74.5–82]	IG: 43.75%; CG: 50%
Vitamin D3 vs. Placebo				
Anweiler C. et al. [55]	Retrospective pre-post cohort study/Elderly outpatients visiting a memory clinic without recent vitamin D supplementation and without prescription of antidementia drugs	IG: *n* = 20; CG: *n* = 24	Median age [IQR],yr: IG: 81.9 [13.2] CG: 75.9 [15.0]	IG: 45%; CG: 45.8%
Vitamin D3 (2000 IU/d) vs. Vitamin D3 (800 IU/d)				
Schietzel S. et al. [60] NCT00599807	RCT (double-blind)/Community-dwelling older adults ≥60 yr with an MMSE ≥ 24 at baseline undergoing elective surgery for unilateral total knee replacement due to severe osteoarthritis	IG: *n* = 137; CG: *n* = 136	IG: 70.2 ± 6.8; CG: 70.5 ± 6.0	IG: 49.6%; CG: 43.4%
Vitamin D3 vs. Placebo				
Aspell N. et al. [51] NCT02804841	RCT (placebo-controlled, double-blind)/Patients ≥60 yr without cognitive impairment (MMSE < 23) and with measured serum vitamin D <125 mmol/L.	IG: *n* = 30; CG: *n* = 29	68.5 ± 4.9	46.7%
Vitamin D + Exercise programs vs. Exercise programs				
Lee Y. et al. [56] KCT0002490	Non-equivalent, control-group experimental study (Pre-test-post-test design)/Adults >65 yr with serum vitamin D levels <20 ng/mL	IG: *n* = 46; CG: *n* = 48	IG: 77.8 ± 6.0; CG: 76.9 ± 6.5	IG: 21.74%; CG: 18.75%
Vitamine D3 vs. No supplementation				
Bischoff-Ferrari H. et al. [58] NCT01745263	RCT (placebo-controlled, double-blind, 2 × 2 × 2 factorial)/Adults ≥70 yr without major health problems in the 5 yr prior to enrollment and MMSE ≥ 24	IG: *n* = 1076; CG: *n* = 1081	IG: 75 ± 4.5; CG: 74.9 ± 4.4	IG: 38%; CG: 38.6%
Vitamin D3 (600 IU/d) vs. Vitamin D3 (2000 IU/d) vs. Vitamin D3 (4000 IU/d)				
Castle M. et al. [59] NCT01631292	RCT (double-blind)/Overweight/obese postmenopausal women with serum 25-hydroxyvitamin D <30 ng/mL	IG_1_: *n* = 15; IG_2_: *n* = 15; IG_3_: *n* = 12;	IG_1_: 58 ± 6.8; IG_2_: 58.5 ± 5.3; IG_3_: 57.2 ± 5.9;	IG_1_: 0%; IG_2_: 0%; IG_3_: 0%;
Vitamin D3 + fish oil supplements vs. Placebo				
Kang J. et al. [53] VITAL trial: NCT01169259. Two substudies: - VITAL-Cog: NCT01669915 - CTSC-Cog(VITAL-DEP): NCT01696435	Large RCT (placebo-controlled, double-blind, 2 × 2 factorial)/People >60 yr free of vascular disease and cancer	VITAL-Cog substudy: IG: *n* = 1710; CG: *n* = 1714 CTSC-Cog substudy: IG: *n* = 396; CG: *n* = 398	VITAL-Cog substudy: IG: 71.9 ± 5.4; CG: 71.8 ± 5.4 CTSC-Cog substudy: IG: 66.9 ± 5.2; CG: 67.3 ± 5.4	VITAL-Cog substudy: IG: 40.9%; CG: 41.4% CTSC-Cog substudy: IG: 48.2%; CG: 51.0%
Vitamin D3 + Calcium carbonate vs. Placebo				
Rossom R.C. et al. [54]	Post-hoc analysis of an RCT (double-blind, placebo-controlled)/Women ≥65 yr without cognitive impairment at baseline	IG: *n* = 2034; CG: *n* = 2109	IG: 70.7; CG: 70.9	IG: 0%; CG: 0%
Fortified yogurts with vitamin D3 and Calcium vs. Non-fortified yogurts				
Beauchet O. et al. [57] NCT02086409	RCT (Unicentre, single-blind, in 2 parallel groups)/Female ≥65 yr with hypovitaminosis D (seum 25 OHD concentration <75 nmol/L), calcemia <2.65 mmol/L and free of dementia	IG: *n* = 20; CG: *n* = 20	IG: 71 ± 3.7; CG: 71.5 ± 5.2	IG: 0%; CG: 0%
Vitamine E (+ selenium) vs. Placebo				
Kryscio R.J. et al. [61] NCT00040378	First an RCT (double-blind, 4 arm); then transformed into a cohort study/men ≥60 yr in absence of dementia	IG_1_: *n* = 1799; IG_2_: *n* = 1881; IG_3_: *n* = 1828; CG: *n* = 1830	IG_1_: 67.5 ± 5.2; IG_2_: 67.6 ± 5.3; IG_3_: 67.6 ± 5.3; CG: 67.3 ± 5.2	IG_1_: 100%; IG_2_: 100%; IG_3_: 100%; CG: 100%
Vitamin E (+ Memantine) vs. Placebo				
Dysken M. et al. [62] NCT00235716	RCT (double-blind, placebo-controlled, parallel-group)/People with mild to moderate AD (MMSE 12–26) currently taking AChEI	IG_1_: *n* = 152; IG_2_: *n* = 155; IG_3_: *n* = 154; CG: *n* = 152	IG_1_: 78.6 ± 7.2; IG_2_: 78.8 ± 7.2; IG_3_: 78.3 ± 7.0; CG: 79.4 ± 7.0	IG_1_: 96%; IG_2_: 96%; IG_3_: 97%; CG: 98%
Vitamin E + Vitamin C vs. Placebo				
Alavi Naeini A.M. et al. [63]	RCT (double-blind, placebo-controlled)/Elderly aged 60–75 yr with MCI and MMSE between 21–26 scores	IG: *n* = 127; CG: *n* = 129	IG: 66.5 ± 0.39; CG: 66.3 ± 0.38	IG: 49.6%; CG: 44.2%

AD: Alzheimer’s disease; AChEI: Acetylcholinesterase inhibitor; CG: control group, DHA: docosahexaenoic acid; IG: intervention group, IG1: intervention group 1, IG2: intervention group 2, IG3: intervention group 3, [IQR]: interquartile range, IU: International unit; MCI: mild cognitive impairment; MMSE: Mini-Mental State Examination; MoCA: Montreal Cognitive Assessment; n: number of participants, RCT: randomized controlled trial, SD: standard deviation, VCIND: vascular cognitive impairment-no dementia; yr: years; 25OHD: 25-hydroxyvitamin D;/d: per day, : male.
